# Chemical Composition, Antioxidant, and Antibacterial Activity of Wood Vinegar from *Litchi chinensis*

**DOI:** 10.3390/molecules21091150

**Published:** 2016-08-30

**Authors:** Jyh-Ferng Yang, Cheng-Hong Yang, Ming-Tsai Liang, Zi-Jie Gao, Yuh-Wern Wu, Li-Yeh Chuang

**Affiliations:** 1 Department of Chemical Engineering and Institute of Biotechnology and Chemical Engineering, I-Shou University, Kaohsiung 840, Taiwan; jfyang@isu.edu.tw (J.-F.Y.); mtliang@isu.edu.tw (M.-T.L.); isu10237005M@cloud.isu.edu.tw (Z.-J.G.); 2 Department of Electronic Engineering, National Kaohsiung University of Applied Sciences, Kaohsiung 807, Taiwan; chyang@cc.kuas.edu.tw

**Keywords:** *Litchi chinensis* wood vinegar, gas chromatography-mass spectrometry, antibacterial activity, antioxidant activity

## Abstract

The antioxidant and antibacterial activities of wood vinegar from *Litchi chinensis*, and its components have been studied. The chemical compositions of wood vinegar were analyzed by gas chromatography-mass spectrometry (GC-MS). A total of 17 chemical compounds were identified, representing 83.96% of the compositions in the wood vinegar. Three major components, included 2,6-dimethoxyphenol (syringol, 29.54%), 2-methoxyphenol (guaiacol, 12.36%), and 3,5-dimethoxy-4-hydroxytoluene (11.07%), were found in the wood vinegar. Antioxidant activities of the acids were investigated from the aspects of 1,1-Diphyl-2-picrylhydrazyl (DPPH) free radicals scavenging capacity, superoxide anion radical scavenging capacity, and reducing power. The pyroligneous acid exhibited high antioxidant activity which was comparable to the reference standards (vitamin C and butylated hydroxyl toluene) at the same dose with IC_50_ values of 36.5 ppm calculated by the DPPH radical scavenging assay, 38.38 g Trolox equivalent/100 g DW by the trolox equivalent antioxidant capacity (TEAC) assay, and 67.9 by the reducing power analysis. Antibacterial activity was evaluated using the disc diffusion and microdilution methods against a group of clinically antibiotic resistant isolates. The major components exhibited broad spectrum inhibition against all the bacterial strains with a range of disc inhibition zoon between 15–19 mm. The minimum inhibition concentration and minimum bactericide concentration against the test strains was ranging in 0.95–3.80 μL/100 μL and 1.90–3.80 μL/100 μL, respectively. Most of the antibiotic resistant strains were more susceptible to the wood vinegar than the non-antibiotic resistant strain except the strain of ornithine resistant *Staphylococcus aureus*. Based on the chemical profile, it was considered that the strongest antioxidant and antibacterial activity of *Litchi chinensis* wood vinegar was due to its highly phenolic compositions. This study revealed that the *Litchi chinensis* wood vinegar is valuable to develop as alternative food antioxidant and antibiotics.

## 1. Introduction

Wood vinegar, also called pyroligneous acid, is a brown, flavorful liquid produced by distillation of wood in the absence of air condition. When the gas generated from combustion is cooled, it condenses into liquid. The carbonization of many different types of wood can be used to produce various wood vinegars including Eucalyptus [1,2], oak [3], bamboo [4], mangrove [5,6], coconut shell [7], and apple trees. Many different sources of wood vinegar have been recognized as safe, natural inhibitors with various bioactivities, which make them suitable for use in antifungal, termiticidal, and repellent applications [8,9,10,11,12]. In addition, wood vinegar exhibits a high degree of antimicrobial activity against various microorganisms [12,13], along with significant antioxidant activity [14,15].

Lignin is known as a class of complex organic polymers that form important structural materials in the formation of cell walls, especially in wood and bark. Lignin makes it possible for the plant′s vascular tissue to conduct water efficiently [16]. Pyrolysis of lignin during the combustion of wood or charcoal are able to provide a range of products, in which the most characteristic ones are methoxy-substituted phenols, such as guaiacol and syringol and their derivatives. In our daily life, these two chemicals provide the main flavor of smoked foods [17].

Gas chromatography mass spectroscopy (GC-MS), a sensitive hyphenated system, is widely used to analyze, identify, and quantify the chemical compositions of natural products. The unknown chemical composition of wood vinegar from different plants can be determined by matching of the peak mass spectra distribution with the NIST MS database [18].

*Litchi chinensis* is a popular fruit produced in bulk in Southern Taiwan. To improve tree development and productivity in the following years, *Litchi* trees must be pruned following each harvest, but most of the trimmings are discarded or burnt as fuel. However, these trimmings could be used to manufacture charcoal, producing wood vinegar as a byproduct. If the resulting wood vinegar possesses active biological properties, it would greatly increase the value of the *Litchi* cultivation.

Although previous studies have indicated that pyroligneous acid has potential as a natural antioxidant and antimicrobial agent, many studies have also reported that wood vinegar produced using different source materials might present different levels of bioactivity and different quantities of bioactive constituents. This study investigates the antioxidant and antibacterial activities of the wood vinegar from *Litchi chinensis*, and determines the chemical composition of the acid, using GC-MS to probe the relationship between the bioactivity and the constituents.

## 2. Results and Discussion

### 2.1. Chemical Profiles of Wood Vinegar From GC-MS

**Figure 1:**
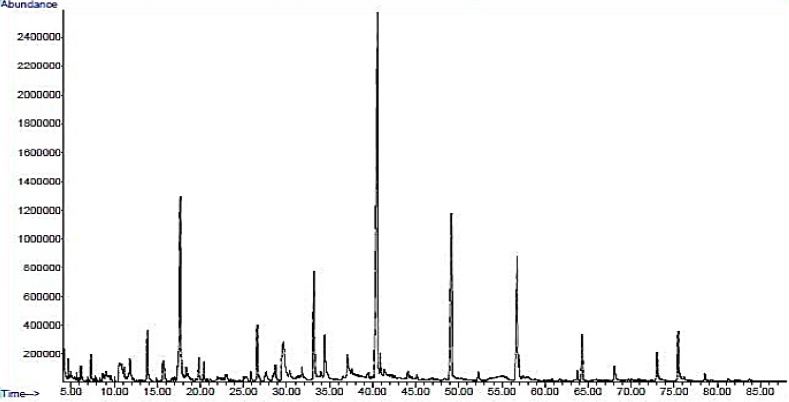
GC-MS analysis of the chemical constituents of the wood vinegar.

**Figure 2:**
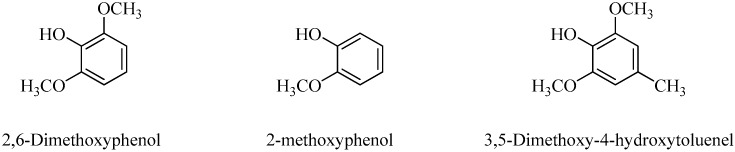
Three major components of the wood vinegar.

The GC-MS analysis of various chemical compositions from the wood vinegar was performed using an Agilent HP-5ms Ultra Inert capillary column (Agilent Technologies, NEW Castle, DE, USA) and total ion chromatogram (TIC) (Agilent Technologies). The absorbance peaks of the chemical compositions from the wood vinegar are shown in Figure 1. The constituents were identified by comparing their mass spectra against the database of NIST MS 14.0 with matches of greater than or equal to 90%. The GC-MS analysis of the wood vinegar revealed a total of 17 different components as listed in Table 1, representing 83.96% of all compositions in the wood vinegar. Among the 17 different compounds, three major components (Figure 2) were 2,6-dimethoxyphenol (syringol, 29.54%), 2-methoxyphenol (guaiacol, 12.36%), and 3,5-dimethoxy-4-hydroxytoluene (11.07%). The minor components were 3-methoxy-1,2-benzenediol (6.12%), catechol (5.17%), creosol (3.15%), 4-ethyl-2-methoxyphenol (3.09%), 3-methyl-1,2-cyclopentanedione (2.65%), and phenol (2.03%). Other components constituted less than 2% of the total yield.

As shown in Table 1, the GC-MS analysis revealed a total of 17 chemical constituents of wood vinegar, most of which were phenol compounds.

**Figure 3:**
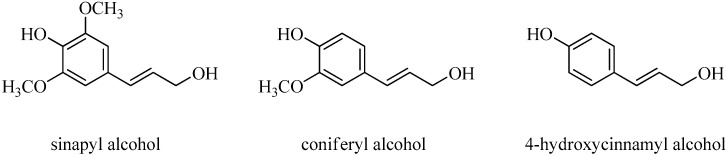
Molecular structures of lignin units.

Lignin is a stable biopolymer assembled from hydroxyphenylpropane (C6–C3) units such as sinapyl alcohol, coniferyl alcohol and 4-hydroxycinnamyl alcohol (Figure 3) [8]. Therefore, phenolic compounds can be obtained from the cleavage of ether and carbon-carbon bonds of lignin.

**Figure 4:**
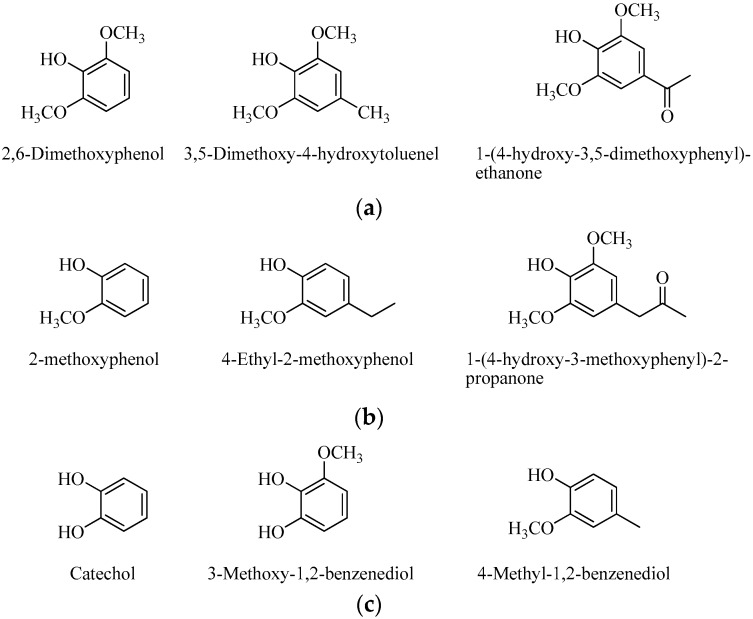
Molecular structures of the phenol compounds from the wood vinegar (**a**) syringol-type; (**b**) guaiacol-type; and (**c**) benzenediol-type.

The phenol compounds identified from the wood vinegar could be classified as syringol-type (42.2%, Figure 4a), including 2,6-dimethoxyphenol (29.54%), 3,5-dimethoxy-4-hydroxytoluene (11.07%), and 1-(4-hydroxy-3,5-dimethoxyphenyl)-ethanone (1.59%), guaiacol-type (16.55%, Figure 4b), including 2-methoxyphenol (12.36%), 4-ethyl-2-methoxyphenol (3.09%), and 1-(4-hydroxy-3-methoxyphenyl)-2-propanone (1.10%), and benzenediol-type (12.59%, Figure 4c), including catechol (5.17%), 3-methoxy-1,2-benzenediol (6.12%), and 4-methyl-1,2,-benzenediol (1.66%).

Among the phenolic components, catechol is probably produced via the demethylation of guaiacol and 3-methoxy-1,2-benzenediol can be obtained from the demethylation of syringol [15]. The results shown in Table 1 indicate that the major lignin units of the wood vinegar were sinapyl alcohol and coniferyl alcohol.

### 2.2. Antibacterial Ability

The results for the in vitro antibacterial properties of the wood vinegar are presented in Table 2. According to the disc inhibition assay, all of the test pathogens were sensitive to the wood vinegar with a range of disc inhibition zone (DIZ) between 15 to 19 mm, indicating that the wood vinegar possesses a broad antibacterial spectrum against different pathogens, with a maximum DIZ (19 mm) against the clinical isolate of *Staphylococcus aureus*, followed by *Acinetobacter baumannii*, *Pseudomonas aeruginosa* and Ornithine resistant *Staphylococcus aureus* with slightly smaller DIZ values (16–17 mm).

The results of minimum inhibitory concentration (MIC) and minimum bactericidal concentration (MBC) determinations showed that the wood vinegar exhibits significant antibacterial activity against *Staphylococcus aureus* and *Pseudomonas aeruginosa*, with respective MIC and MBC values of 0.95–1.90 μL/100 μL and 1.90 μL/100 μL. With the exception of ornithine-resistant *Staphylococcus aureus* (ORSa), most of the tested antibiotic-resistant strains showed moderate antibacterial activity against the standard strain (ATCC 25257), a non-antibiotic resistant strain, with respective MIC and MBC values of 2.38–2.86 μL/100 μL and 2.86 μL/100 μL.

Chan recently reported that *Matang* wood vinegar displayed potent antibacterial activity [18] against the strains of Gram-positive *Bacillus cereus*, *Micrococcus luteus* and *Staphylococcus aureus*, and Gram-negative *Escherichia coli*, *Salmonella typhi*, and *Pseudomonas aeruginos*a. Based on their MIC determination results, the overall ranking of susceptibility was *B. cereus* > *M. luteus* ~ *S. aureus* ~ *P. aeruginosa* > *E. coli* ~ *S. typhi*. Their results are consistent to our finding that the Gram-positive strain *S. aureu*s was the most susceptible, while the Gram-negative *E. coli* showed only light susceptibility to the wood vinegar. Many previous reports suggest that Gram-positive bacteria are generally more susceptible to plant extracts than Gram-negative bacteria because of the absence of an outer membrane of lipoprotein and lipopolysaccharide. An outer membrane of lipoprotein and lipopolysaccharide is selectively permeable and can regulate access of antimicrobials into the underlying cell structures [19]. However, the Gram-positive strain ORSA showed the lowest susceptible to the wood vinegar, and it is presumed that strong biofilm formers (ORSA) might result in stronger drug resistance [20]. Other wood vinegar sources including bamboo, eucalyptus, and rubber also exhibited antimicrobial activity against dermatitis, environment, and plant bacteria, as well as fungi [21]. A concentration of less than 10% is suggested as suitable for use as an antimicrobial agent while preventing leaf burning and some pathogenic fungi on PDA.

More than 200 chemical constituents of pyroligneous acid have been identified from different resources [1,2,3,4,5,6,7,8,9]. Earlier reports showed that the major components of organic acids and phenolics in wood vinegar can inhibit pathogenic fungi and bacteria [10,11,12,13]. The chemical profile presented in Table 1 shows that more than 70% of all wood vinegar compositions were identified as phenolic compounds, of which three major components exhibit antibacterial activity: 2,6-dimethoxyphenol (Syringol, 29.54%), 2-methoxyphenol (guaiacol, 12.36%), and 3,5-dimethoxy-4-hydroxytoluene (11.07%). Therefore, we conclude that the three major phenolics and other minor organic acids contribute to the antibacterial activities of the wood vinegar from *Litchi chinensis*.

### 2.3. Antioxidant Assay

The antioxidant assay results are shown in Table 3. In the DPPH radical scavenging ability assay, the DPPH IC_50_ value (the concentration required to scavenge DPPH radical by 50%) of wood vinegar was about 36.53 ppm (Table 3). The DPPH IC_50_ values of butylated hydroxyl toluene (BHT) and vitamin C were about 175 ppm and 7 ppm, respectively. The result indicates that the radical scavenging ability of wood vinegar is higher than the commercial chemical antioxidant (BHT), but lower than the natural antioxidant (vitamin C). The reducing power analysis found that the reducing power increased with the concentration of each sample. The ranking order for reducing power was wood vinegar (67.9 abs/10^−3^ ppm) > Vitamin C (7.3 abs/10^−3^ ppm) > BHT (2.2 abs/10^−3^ ppm). Significantly, the wood vinegar exhibited higher reducing power than the commercial antioxidants (BHT and vitamin C). For the Trolox equivalent antioxidant capacity (TEAC) assay, the wood vinegar (38.38 g Trolox/100 g DW) and Vitamin C (38.47 g Trolox/100 g DW) showed similar TEAC values that were both higher than that of BHT (35.64 g Trolox/100 g DW). The TEAC assay results indicate that wood vinegar possesses antioxidant ability similar to that of vitamin C and higher antioxidant capacity than BHT. The total phenolic and flavonoid contents in the wood vinegar were respectively 37.34 g gallic acid/100 g DW and 4.42 g quercetin/100 g DW (Table 3). These results imply that the wood vinegar contains a high quantity of phenolic compounds and flavonoids, which is consistent with the results obtained from GC-MS analysis.

Previous studies have shown that phenolic compounds exhibit strong free radical scavenging capability, reducing power, and antioxidant capability and can, therefore, be used as reductants and antioxidants. Plant phenolic and flavonoid components are widely distributed in the tissues of plants, and also play a pivotal role in highly effective bioactivity. Due to the side effects of chemical synthetic antioxidants (such as BHT and BHA), more attention is now focused on the antioxidant activity of phenolic compounds from plants. Previous reports have verified that the pyroligneous acid exhibits significant antioxidant activity. Ma et al. [22] found that the fruit of *S. chinensis* exhibits superoxide anion scavenging activity and antioxidant activity. Loo et al. [5,6] demonstrated that pyroligneous acid from the mangrove plant and *Rhizophora apiculate* exhibit antioxidant and free radical scavenging activities. All these results indicate that pyroligneous acid could potentially be a natural antioxidant.

Many studies have indicated that pyroligneous acid is rich in phenolic compounds [10,11,12], which are pyrolytic products of lignin and hemicellulose, comprising 30%–60% of the total organic compounds in the acid. 2,6-dimethoxyphenol, also known as syringol, has been identified as having a woody/herby flavor and smoky odor [23]. Syringol exhibited antioxidant activities based on its DPPH radical scavenging activity, ABTS radical cation scavenging activity, phosphomolybdenum and ferric reducing antioxidant power [6]. In addition, 3-Methyl-1,2-cyclopentanedione may be regarded as a potent regulator of (ONOO­)-mediated diseases via direct scavenging of the reactivity of ONOO­ and can prevent (ONOO­)-induced damage of GSH reductase [24]. Loo et al. reported the isolation of three antioxidative compounds (syringol, catechol, and 3-methoxycatechol) from mangrove wood vinegar [5] which is consistent to our finding in the *Lichi* wood vinegar. All of these results indicate that the pyroligneous acid from *Lichi* has potential to be developed as a natural antioxidant.

## 3. Materials and Methods

### 3.1. Materials and Chemicals

The raw wood vinegar was prepared from *Litchi chinensis* using a traditional Japanese black charcoal kiln and collected by using running water through a shuttle (7–10 in length), which connected with the chimneys, to condense the smoke. Pyroligneous acids were collected from a temperature range of 100–600 °C and prepared by the Bu-Quang charcoal company (Kaohsiung, Taiwan). The raw wood vinegar was stored in the dark at 4 °C for analysis. All chemicals (1,1-diphyl-2-picrylhydrazyl (DPPH), butylated hydroxytoluene (BHT), trichloroacetic acid (TAC), ascorbic acid, Folin Ciocalteu’s reagent, potassium ferricyanide, ferric chloride, sodium carbonate, and all analytical chemicals) were purchased from Sigma (Sternheim, Germany). The clinical antibiotic resistant strains (*Staphylococcus aureus* 985, *Acinetobacter baumanni*i 814, *Pseudomonas aeruginos*a 717, and ornithine-resistant *Staphylococcus* *aureus* 220) used in this research were isolated from blood and phlegm samples provided by Chia-Yi Christian Hospital (Chia-Yi, Taiwan). The standard strain (*Escherichia coli* ATCC 25257) was purchased from the Taiwan′s Food Industry Research and Development Institute Bioresources Collection and Research Center (Hsinchu, Taiwan).

### 3.2. Instrument

Analysis used an Agilent 7890B gas chromatography instrument, combined with an Agilent-5977A mass spectrometer (Agilent Technologies) equipped with electron ionization (EI) and quadrupole analyzer, and an Agilent Chem Station data system. GC separation was performed on a 30 m HP-5ms Ultra Inert capillary column with an internal diameter of 0.25 mm and a film thickness of 0.25 μm (Agilent 19091S-433UI, Agilent Technologies).

### 3.3. Methods

#### 3.3.1. Phytochemical Composition Analysis

The components of wood vinegar were subjected to GC-MS analysis on an Agilent system consisting of a model 7890B gas chromatographer and a model 5977A mass selective detector (MSD, electron energy, 70 eV). The carrier gas was helium (99.99%) with a flow rate of 0.8 mL/min. The injector and detector temperatures were respectively set at 250 °C and 250 °C. Spectra were obtained over a scan range of 50 to 550 amu at 2 scans/s. The GC program was set as follows: the initial temperature was 60 °C and held for 10 min, then increased by 2 °C/min to 80 °C and held for 5 min, then raised by 2 °C/min to 110 °C and held 6 min, then raised by 2 °C/min to 120 °C and held for 5 min, and finally raised by 2 °C/min to 180 °C and held at 180 °C for 2 min. The wood vinegar (1.0 μL) was injected automatically while maintaining a solvent delay of 4 min. Interpretation of the mass spectrum was made by comparing the peak distribution against the database of National Institute Standard and Technology (NIST MS 14.0, Gaithersburg, MD, USA). Relative percentages of the chemical compositions were calculated based on the GC peak areas without correction.

#### 3.3.2. Antibacterial Activity Assay

##### Disc Inhibitory Assay

A Petri dish was prepared with a base layer of Muller Hinton (MH) agar (10 mL) and a top layer of 0.75% MH agar (5 mL), then inoculated with 50 μL of each bacterial suspension (10^5^ CFU/mL). Paper discs (6 mm in diameter) were impregnated with 30 μL of wood vinegar and placed on the inoculated plates, then incubated at 37 °C for 14 h. The diameters of the inhibition zones (DIZ) were measured [25].

##### Determination of Minimum Inhibitory Concentration (MIC) and Minimum Bactericidal Concentration (MBC)

A broth dilution method was used to determine the minimum inhibitory concentration (MIC) and minimum bactericidal concentration (MBC) [25]. The wood vinegars (50, 100, 125, 150, and 200 μL) were diluted in 5 mL LB broth. Each strain (10^6^ CFU/mL) was added and incubated at 37 °C at 220 rpm for 16 h. Subsequently, 100 μL of the culture mixture was coated on the MH agar medium, and the colony numbers were counted after incubating for 16 hrs at 37 °C. The MIC is defined as the concentration level that can inhibit 90% of bacteria development, with 99% bacterial inhibition set as the MBC.

#### 3.3.3. Determination of Total Phenolic Content

The total phenolic content of wood vinegar was determined using the Folin-Ciocalteu method using gallic acid as a standard [26]. Briefly, 0.1 mL of wood vinegar was added into 0.1 mL of 25% Folin-Ciocalteu reagent and 2 mL of 2% Na_2_CO_3_ solution with thorough shaking. After reacting at room temperature for 30 min, the absorbance was measured at 760 nm and the phenolic content was calculated according to the equation that was obtained from the standard gallic acid.

#### 3.3.4. Determination of Flavonoids Content

The total flavonoid content of wood vinegar was determined using the method described as our previous study [26] with slight modifications. Briefly, one mL of the solution containing 1 mg/mL wood vinegar with methanol was added into 0.1 mL of 10% aluminium nitrate, 0.1 mL of 1 M potassium acetate and 3.8 mL of methanol. After reacting at room temperature for 40 min, the absorbance was read spectrophotometrically at 415 nm, using quercetin as a standard. The concentrations of flavonoid compounds were calculated using the equation obtained from the standard quercetin graph.

#### 3.3.5. Antioxidant Activity Assay

##### DPPH Free Radical Scavenging Ability

The DPPH free radical scavenging ability of wood vinegar was evaluated using the method described as our previous study [26] with some modifications. Briefly, one mL of various concentrations of wood vinegar in methanol was added to 0.25 mL of DPPH solution in methanol. The mixture was shaken and allowed to stand for 30 min in room temperature. The absorbance of the resulting solution was then measured at 517 nm with a spectrophotometer. The percentage of DPPH free radical inhibition was calculated with the following equation:

(1) $$\mathrm{DPPH}\text{ Inhibition (\%) }=\;\left(1-\frac{\text{Abs. of Exp.}-\text{Abs. of Comp. }}{\text{Abs. of control }}\right)\times \text{100\%}$$

##### Reducing Power

The reducing power of wood vinegar was determined using the method described as our previous study [26] with some modifications. Briefly, 150 μL of wood vinegar in methanol was mixed with 150 μL of 200 mM sodium phosphate buffer (pH 6.6) and 150 μL of 1% K_3_Fe(CN)_6_. The mixture was then incubated at 50 °C for 20 min. Subsequently, 150 μL of 10% trichloroacetic acid, 600 μL of double deionized water, and 600 μL of FeCl_3_ were added and the mixture was incubated for a further 14 min. The absorbance of each reaction mixture was measured at 700 nm. A higher absorbance indicates a higher reducing power.

##### Trolox Equivalent Antioxidant Capacity

The Trolox equivalent antioxidant capacity (TEAC) of wood vinegar was determined as our previous study [26] with some modifications. In brief, 20 μL of various concentration of wood vinegar in methanol was mixed with 1 mL of 0.175 mM ABTS solution, and the mixture was shaken and allowed to stand for 10 min at room temperature. The absorbance of the resulting solution was then measured at 734 nm with a spectrophotometer. Percentage of ABTS radical inhibition was calculated with the following equation:

(2) $$\text{Inhibition (\%) }=\;\left[\text{1 }-\;\left(\frac{\text{Abs. of sample}}{\text{Abs. of control}}\right)\times 100\%\right]$$

The TEAC value was calculated with the following equation:

(3) $$\text{TEAC }=\;\left(\frac{{\text{IC}}_{50}\text{ of trolox}}{{\text{IC}}_{50}\text{ of sample }}\right)$$

#### 3.3.6. Statistical Analysis

Data were expressed as mean and standard deviation (SD). A one way variance was used to analyze data, with *p* < 0.05 representing a significant difference between means (Duncan’s multiple range test).

## 4. Conclusions

This is the first study of the chemical composition, and antibacterial and antioxidant activities of wood vinegar from *Litchi chinensis*. The results reveal that *Lichi* wood vinegar contains seventeen compounds in various concentrations. More than 70% of the components were identified as phenolic compounds which could be classified into the syringol-type (42.2%), guaiacol-type (16.55%), and the benzenediol-type (12.59%). Antioxidant activity assay results showed significant antioxidant activity comparable to commercial antioxidants. Moreover, the wood vinegar showed significant antibacterial activity against clinical antibiotic-resistant pathogens, which implies they can be developed into useful sterile products for medical, aquaculture, and livestock breeding applications. The results suggest the antioxidant and antibacterial activities of *Litchi chinensis* wood vinegar are contributed by the three major phenolic compounds, 2,6-dimethoxyphenol (Syringol, 29.54%), 2-methoxyphenol (guaiacol, 12.36%), and 3,5-dimethoxy-4-hydroxytoluene (11.07%). The chemical constituents of the wood vinegar must be identified to maximize utilization.

## Figures and Tables

**Table 1:** Chemical constituents of the wood vinegar analyzed by GC-MS.

No.	RT (min)	Name of the Compounds	Molecular Formula	MolecularWeight	% Area
1	4.644	2-methyl-pyridine	C_6_H_7_N	93.06	0.23
2	10.664	Phenol	C_6_H_6_O	94.04	2.03
3	13.869	3-methyl-1,2-cyclopentanedione	C_6_H_8_O_2_	112.05	2.65
4	15.682	2-methylphenol	C_7_H_8_O	108.06	1.54
5	17.663	2-methoxyphenol (Guaiacol)	C_7_H_8_O_2_	124.05	12.36
6	19.838	Maltol	C_6_H_6_O_3_	126.03	1.02
7	20.416	3-ethyl-2-hydroxy-2-cyclopenten-1-one	C_7_H_10_O_2_	126.07	0.74
8	26.601	Creosol	C_8_H_10_O_2_	138.07	3.15
9	29.641	Catechol	C_6_H_6_O_2_	110.04	5.17
10	33.199	3-methoxy-1,2-benzenediol	C_7_H_8_O_3_	140.05	6.12
11	34.415	4-ethyl-2-methoxyphenol	C_9_H_12_O_2_	152.08	3.09
12	37.068	4-methyl-1,2-benzenediol	C_7_H_8_O_2_	124.05	1.66
13	40.568	2,6-dimethoxyphenol (Syringol)	C_8_H_10_O_3_	154.06	29.54
14	40.895	3,4-dimethoxyphenol	C_8_H_10_O_3_	154.06	0.93
15	49.159	3,5-dimethoxy-4-hydroxytoluene	C_9_H_12_O_3_	168.08	11.07
16	56.966	1-(4-hydroxy-3-methoxyphenyl)-2-propanone	C_10_H_12_O_3_	180.08	1.10
17	73.001	1-(4-hydroxy-3,5-dimethoxyphenyl)-ethanone	C_10_H_12_O_4_	196.07	1.59

**Table 2:** Antibacterial activity of the wood vinegar.

Strains *	Disc Inhibition Zone (mm) ^#^		MIC (μL/100 μL)	MBC (μL/100 μL)
	Wood Vinegar	Tetracycline (7.5 mg/mL)		
Ec 25257	15.20 ± 0.40	27.90 ± 0.00	2.38–2.86	2.86
Ab 814	17.50 ± 0.20	12.73 ± 0.53	1.90	2.38
Sa 985	19.00 ± 1.00	17.09 ± 0.09	0.95–1.90	1.90
Pa 717	17.70 ± 0.20	23.34 ± 1.91	0.95–1.90	1.90
ORSa 220	16.30 ± 1.10	21.31 ± 0.11	2.38–3.80	3.80

* Ec: *Escherichia coli*; Ab: *Acinetobacter baumannii*; Sa: *Staphylococcus aureus*; Pa: *Pseudomonas aeruginosa*; ORSa: Ornithine-resistant *Staphylococcus aureus* ^#^: mean ± SD.

**Table 3:** Antioxidant activity of the wood vinegar ^#^.

Samples	DPPH IC_50_ (ppm)	TPC (g Gallic acid/100 g DW)	TFC (g Quercetin/100 g DW)	TEAC (g Trolox Equivalent/100 g DW)	Reducing Power (abs/10^−3^ ppm)
Wood vinegar	36.53 ± 1.57	37.34 ± 0.07	4.42 ± 0.01	38.38 ± 0.12	67.9
BHT	175.12 ± 19.92	-	-	35.64 ± 0.35	2.2
Vitamin C	7.01 ± 0.61	-	-	38.47 ± 0.04	7.3

BHT: butylated hydroxyl toluene; DPPH: DPPH free radical scavenging ability; TPC: total phenolic content; TFC: total flavonoid content; TEAC: trolox equivalent antioxidant capacity; -: undetected ^#^: mean ± SD.
